# Cardanol Groups Grafted on Poly(vinyl chloride)—Synthesis, Performance and Plasticization Mechanism

**DOI:** 10.3390/polym9110621

**Published:** 2017-11-15

**Authors:** Puyou Jia, Meng Zhang, Lihong Hu, Rui Wang, Chao Sun, Yonghong Zhou

**Affiliations:** 1 National Engineering Lab for Biomass Chemical Utilization, Key Lab on Forest Chemical Engineering, State Forestry Administration, and Key Lab of Biomass Energy and Materials, Institute of Chemical Industry of Forest Products, Chinese Academy of Forestry (CAF), 16 Suojin North Road, Nanjing 210042, China; jiapuyou@icifp.cn (P.J.); hlh@icifp.cn (L.H.); 2 Institute of New Technology of Forestry, Chinese Academy of Forest (CAF), Beijing 100091, China; 3 College of Materials Science and Engineering, Nanjing Forestry University, 159 Longpan Road, Nanjing 210037, China; by861209@126.com (R.W.); sc930510@163.com (C.S.)

**Keywords:** cardanol, plasticizer, poly(vinyl chloride), migration, plasticization mechanism

## Abstract

Internally plasticized poly(vinyl chloride) (PVC) materials are investigated via grafting of propargyl ether cardanol (PEC). The chemical structure of the materials was studied by FT-IR and ^1^H NMR. The performace of the obtained internally plasticized PVC materials was also investigated with TGA, DSC and leaching tests. The results showed that grafting of propargyl ether cardanol (PEC) on PVC increased the free volume and distance of PVC chains, which efficiently decreased the glass transition temperature (*T*_g_). No migration was found in the leaching tests for internally plasticized PVC films compared with plasticized PVC materials with commercial plasticizer dioctyl phthalate (DOP). The internal plasticization mechanism was also disscussed according to lubrication theory and free volume theory. This work provides a meaningful strategy for designing no-migration PVC materials by introducing cardanol groups as branched chains.

## 1. Introduction

Plasticizer is one of the most important plastic additives, and is used to improve processability, plasticity and flexility of plastics. The most widely used plasticizers are phthalate esters, which account for 80% of the total consumption of plasticizer [1]. However, the potential toxicity of these phthalate esters to the human body has been reported, which has led to their restriction in consumer products [2,3,4]. In order to reduce the toxicity of plasticzier, many environmentally friendly plasticizers have been investigated, such as epoxidized jatropha oil [5], cardanol derivatives [6], polymer-plasticizer [7], polyol ester [8] and phosphate plasticizer [9]. However, these alternative plasticizers will migrate from plastic products with the prolongation of aging time, thus shortening the life of the products. To avoid the migration of plasticizer, Navarro et al. and Lee et al. [10,11,12] have studied an internal plasticization strategy whereby phthalate-based thiol additives and hyperbranched polyglycerol, respectively, were grafted onto the polymer matrix. The glass transition temperature (*T*_g_) of PVC materials grafting hyperbranched polyglycerol groups was kept below 0 °C. Propargyl ether triethyl citrate and castor oil-based derivative were covalently bonded to the polymer matrix as an internal plasticizer, as reported by Jia et al. [13,14]. These studies indicated that this strategy was efficient for producing flexible polymer materials, and avoiding the migration of plasticizer.

Cardanol is one of the most favorable biomass resources for plasticizer production due to its relatively low cost and similar chemical structure to conventional phthalate plasticizers such as dioctyl phthalate (DOP). Cardanol derives from an agricultural by-product that is abundantly available in many parts of the chemical industry [15,16]. There are some reactive groups on the chemical structure of cardanol, such as the unsaturated carbon chains on the branched chains, benezene ring and hydroxy groups. These groups can occur hydrogenation, polymerization, sulfuration, esterification and epoxidation reactions. [17]. Therefore, the strategy of grafting cardanol groups grafted on PVC matrices is expected to produce plasticized PVC materials without migration. Recently, Po et al. studied an approach that covalently linked cardanol to the PVC matrix via Click reaction, but the internal plasticization mechanism was not discussed in detail in terms of traditional plasticization theory [18]. A kind of internally plasticized PVC material was prepared via replacing chlorine using the mannich base of cardanol butyl ether in 2017 [19], but the plasticizing efficiency of the method was lower than for the Click reaction [10,12]. 

Inspired by the above-mentioned pioneer works, we herein report a strategy for developing internally plasticized PVC materials by grafting propargyl ether cardanol onto the PVC matrix. Specifically, different mass of cardanol groups were grafted onto PVC matrices to obtain no-migration, flexible PVC materials. Differential scanning calorimetry (DSC) measurements were used to detect the plasticizing efficiency of the strategy, and to compare it with pure PVC. The migration stability of the internally plasticized PVC materials in n-hexane was investigated. For comparison, the migration stability of PVC films plasticized with the traditional plasticizer DOP was also tested. In addition, the internal plasticization mechanism was discussed in terms of traditional plasticization theories, such as free volume theory and lubrication theory. 

## 2. Materials and Methods 

### 2.1. Materials

Cardanol (99%, acid value 5.5–6.6. Iodine value 210–250) was provided by Jining Hengtai Chemical Co., Ltd. (Jining, China) Propargyl bromide solution, tetrahydrofuran (THF), potassium carbonate, sodium azide, methanol, acetone, cuprous bromide, 5,5-dimethyl-2,2-dipyridyl, *N*,*N*-dimethylformamide (DMF) and dioctyl phthalate (DOP) were kindly provided by Nanjing Chemical Reagent Co., Ltd. (Nanjing, China) Polyvinyl chloride (PVC) was supplied by Hanwha (KM-31, Seoul, Korea).

### 2.2. Synthesis of Propargyl Ether Cardanol 

To a 100 mL flask equipped with a condenser tube was added 30.4 g (100 mmol) of cardanol, 13.08 g (110 mmol) of propargyl bromide solution and 15.2 g (110 mmol) of potassium carbonate in 50 mL of acetone. The mixture was stirred at 65 °C for 12 h. The solution was purified by evaporating under vacuum after washing the mixture with deionized water (yield: 97%).

### 2.3. Synthesis of Azide-Functionalized PVC (PVC-N_3_)

**Figure 1:**
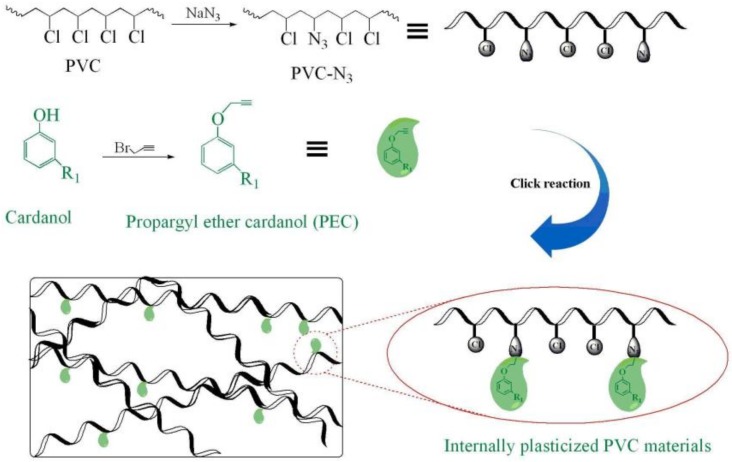
Synthesis of internally plasticized PVC materials.

To a 100 mL flask were added 2.0 g of PVC, 2.0 g of NaN_3_ and 100 mL of DMF. The mixture was allowed to stir at 30 °C for 24 h and precipitated into water/methanol mixture (1/1 by volume), and dried in a vacuum to obtain the PVC–N_3_ [14]. Figure 1 showed the synthetic route of PVC–N_3_. Elemental analysis: 35.13% C, 8.21% H, 18.47% N, and 38.19% Cl.

### 2.4. Synthesis of Internally Plasticized PVC Materials

Internally plasticized PVC materials were prepared by dissolving a certain amount of PVC–N_3_, PEC, cuprous bromide and 2,2′-dipyridine in 40 mL of DMF in a three-neck flask equipped with a mechanical stirrer, nitrogen pipe and thermometer. The reaction was kept at 30 °C and stirred for 24 h. Then, the mixture was precipitated into water/methanol mixture (1/1 by volume) after filtering to remove the copper salts, and dried in a vacuum to obtain PVC-0.25PEC. Table S1 (see Supplementary Materials) shows the composition of reactants. Figure 1 shows the synthesis route. 

### 2.5. Preparation of PVC Films and PVC–DOP Films

A total of 1 g of internally plasticized PVC materials were dissolved in 20 mL of THF. The mixture was stirred at 40 °C for 20 min until the solution appeared transparent, and was then poured into a glass petri dish (5 cm diameter), and dried in a constant temperature drying box at 60 °C for 24 h to completely remove residual THF. PVC–DOP films were prepared by dissolving a total of 2 g of PVC and 1 g of DOP in 20 mL of THF using the same method. 

### 2.6. Characterization

#### 2.6.1. Elemental Analysis

Elemental analysis was conducted on an elemental PE-2400 analyzer (PERKINELMER Instrument Crop., Waltham, MA, USA).

#### 2.6.2. Fourier-Transform Infrared (FT-IR)

Fourier transform infrared (FT-IR) spectra of propargyl ether cardanol and internally plasticized PVC materials were investigated on a Nicolet iS10 FTIR measurement (Nicolet Instrument Crop., Madison, WI, USA). The spectra were acquired in the range of 4000 to 500 cm^−1^ at a resolution of 4 cm^−1^.

#### 2.6.3. ^1^H Nuclear Magnetic Resonance (NMR)

^1^H NMR measurements were conducted on an AV-300 NMR spectrometer (Bruker Instrument Crop., Karlsruhe, Germany) at a frequency of 400 MHz. CDCl_3_ was used as solvent and tetrametnylsilane (TMS) as an internal standard.

#### 2.6.4. Gel Permeation Chromatography (GPC)

The molecular weights of PVC and internally plasticized PVC materials were measured using an efficient gel chromatograph made by Waters, Milford, MA, USA at 30 °C (flow rate: 1 mL/min, column: mixed PL gel 300 mm × 718 mm, 25 μm) using THF as solvent. The number-average molecular weight, weight-average molecular weight and polydispersity indices (*M*_w_/*M*_n_) were calculated by calibrating with polystyrene standards. 

#### 2.6.5. Thermogravimetric Analysis (TGA)

TGA was carried out using a TG209F1 TGA thermal analysis instrument (Netzsch Instrument Corp., Bavaria, Germany) in N_2_ atmosphere (50 mL/min) at a heating rate of 10 °C/min. The temperature was scanned from 40 to 600 °C.

#### 2.6.6. Differential Scanning Calorimetry (DSC)

Glass transition temperature (*T*_g_) was characterized using a NETZSCH DSC 200 PC analyzer (Bavaria, Germany); the temperature ranged from −60 °C to 100 °C in N_2_ atmosphere (50 mL/min) at a heating rate of 20 °C/min. *T*_g_ values reported were taken from the second heating run in order to eliminate thermal history and to correspond to the midpoint of the DSC curves measured from the extension of the pre- and post-transition baseline. 

#### 2.6.7. Leaching Tests

PVC films and internally plasticized PVC films were cut so that different groups had the same surface area. The thickness of all the films was around 2.0 mm. The films were weighted and immersed in n-hexane at 50 °C for 2 h. Then, these films were dried and reweighed. The extraction loss was calculated according to Equation (1).

Degree of migration = (*W*_1_ − *W*_2_)/*W*_1_ × 100 (1)

where *W*_1_ = initial weight of test films, and *W*_2_ = final weight of test PVC films [20].

## 3. Results

### 3.1. Chemical Structure of PEC

Figure S1 presents the FT-IR spectra of the cardanol and PEC. The strong and broad absorption peak at 3332 cm^−1^ in the FT-IR spectra of the cardanol was attributed to stretch vibration of –OH groups. The absorption peak appeared at 3007 cm^−1^, which was assigned to the olefinic C–H stretch of cardanol. The peaks at 2924 and 2853 cm^−1^ were associated with the =C–H and –C–H bonds, respectively. The peak at 1586 cm^−1^ was attributed to the C–C bonds. The broad absorption of the C=C stretching vibration mode from the benzene ring could be observed in the range of 1454–1152 cm^−1^ [20,21]. In comparison to the FT-IR spectra of the cardanol, the characteristic absorption peak of the –OH groups at 3007 cm^−1^ disappeared in the FT-IR spectra of PEC. A new peak appeared at 3306 cm^−1^, which was attributed to alkyne C–H stretch, and the C≡C stretch characteristic absorption peak appeared at 2122 cm^−1^.

^1^H NMR of cardanol and PEC was also investigated. In ^1^H NMR of cardanol (Figure S2, see Supplementary Materials), the signal appeared at δ 0.94 ppm, which was assigned to the protons of the methyl groups on the branched chains. Strong signals appeared at δ 1.36, δ 1.41, δ 2.09, δ 2.5 and δ 2.8 ppm, and were attributed to the protons of methylene groups. Peaks appeared at δ 5.41 ppm, which were associated with protons of hydroxyl groups. The signal of the olefin groups appeared at δ 5.44 ppm, and the signals of the protons for the benzene rings could be observed at δ 6.71, δ 6.82 and δ 7.18 ppm [19,21,22]. In comparison to the ^1^H NMR spectrum of cardanol, a new signal appeared at δ 4.75 ppm, which was attributed to the protons of methylene groups connected to alkynyl groups. Another new peak appeared at δ 2.61 ppm, which was associated with the protons of alkynyl groups. The FT-IR and ^1^H NMR data indicated that PEC had been obtained.

### 3.2. Chemical Structure of Internally Plasticized PVC Materials

**Figure 2:**
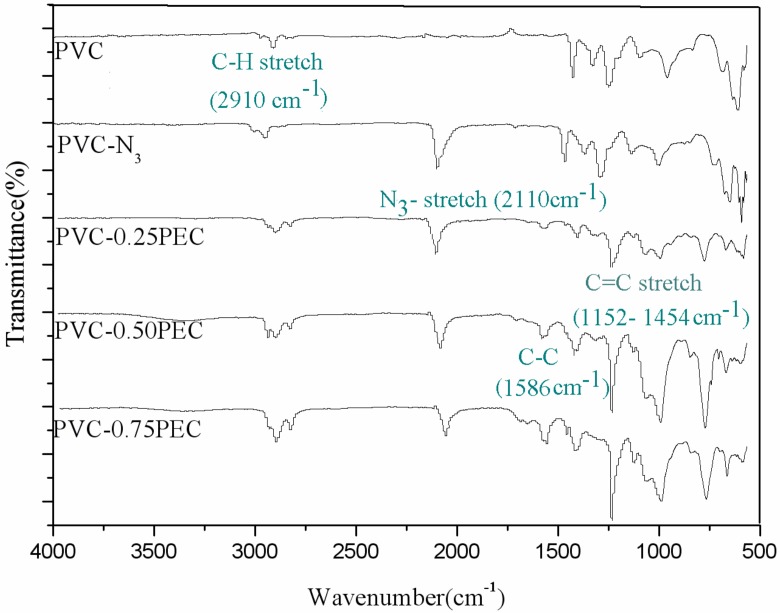
FT-IR spectra of PVC and internally plasticized PVC materials.

Figure 2 shows the FT-IR spectra of PVC and PVC-0.25PEC, PVC-0.50PEC and PVC-0.75PEC. In comparison to the FT-IR spectra of PVC, a new strong peak appeared at 2110 cm^−1^, which was attributed to the characteristic absorption peak of the azide group (–N_3_ stretch) [23,24,25], indicating that PVC–N_3_ had been prepared. With more PEC grafted onto the PVC matrix, the peaks at 2910, 1586 and 1152–1454 cm^−1^, which were attributed to C–H stretch, C–C bonds, and the C=C stretching vibration mode derived from the benzenoid ring of PEC, respectively, appeared stronger than PVC–N_3_, indicating that the PEC had been grafted onto the PVC matrix. 

**Figure 3:**
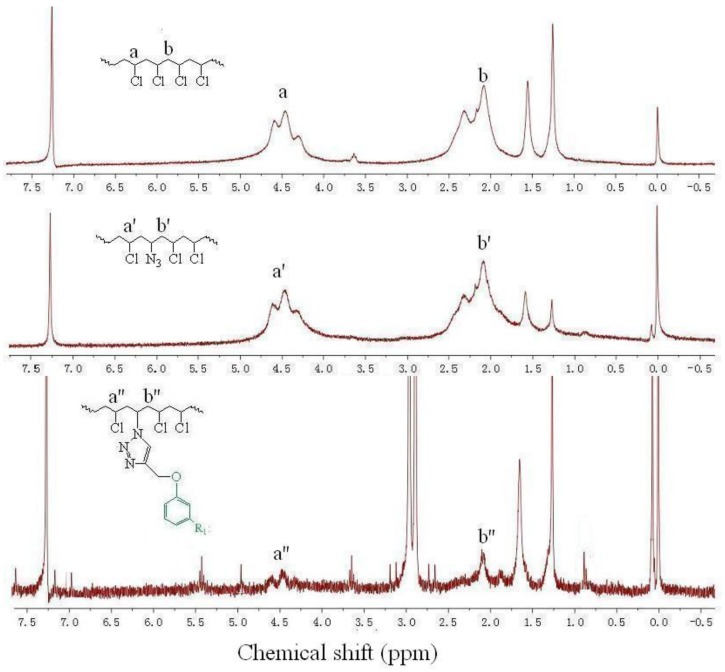
^1^H NMR of PVC and internally plasticized PVC materials.

^1^H NMR of PVC and internally plasticized PVC materials are presented in Figure 3. As can be seen from the ^1^H NMR spectra of PVC, peak at δ 4.5 ppm was attributed to protons of –C*H*Cl–(CH_2_)– [14,18,26]. Peak b at δ 2.2 ppm was assigned to protons of –CHCl–(C*H*_2_)–. The ^1^H NMR of PVC–N_3_ was similar to PVC, indicating that the chemical environment of the protons was similar. Peak a′ at δ 4.5 ppm was associated with the protons of –C*H*Cl–(CH_2_)–, and peak b′ at δ 2.2 ppm was attributed to the protons of –CH(N_3_)–(C*H*_2_). Peaks a′′ and b′′ at δ 4.5 and 2.2 ppm were associated with the protons of –C*H*Cl–(CH_2_)– and –CH(N_3_)–(C*H*_2_)–, respectively, which were weaker than for PVC and PVC–N_3_, but some new peaks appeared at δ 7.18, δ 5.5, δ 2.85 and δ 0.89 ppm, which were associated with the protons of benzene rings, olefin groups, methylene groups and methyl groups of branched chains derived from PEC [26,27,28], respectively, indicating that the PEC had been grafted onto the PVC matrix.

**Figure 4:**
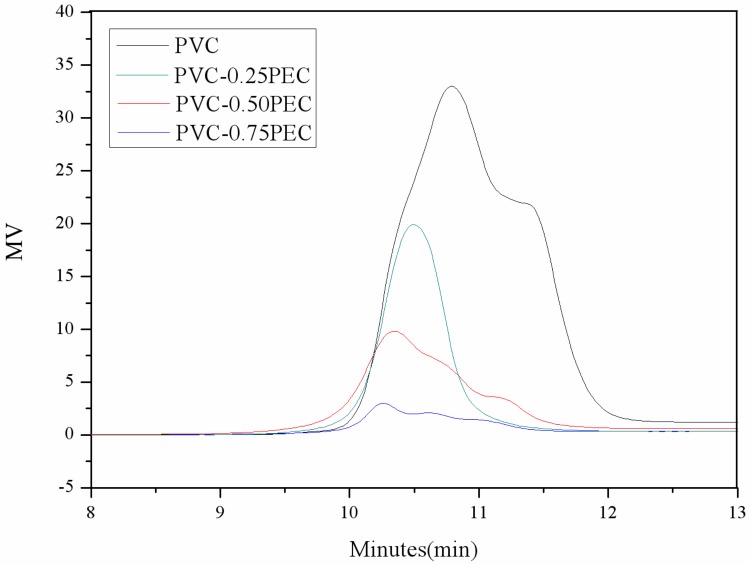
GPC spectra of PVC and internally plasticized PVC materials.

The click reaction level can be evaluated by examining the molecular weight change [29,30]. The molecular weight and polydispersity indices of PVC and internally plasticized PVC materials were investigated by GPC analysis. The results are shown in Figure 4 and Table 1. As can be seen in Figure 4, the GPC peak of internally plasticized PVC materials shows a clear shift to a higher molecular weight region compared to that of PVC, which indicates that the cardanol groups had been grafted onto the poly (vinyl chloride) via click reaction. PVC and PVC-0.25PEC show a single GPC peak with a clear shift to a higher molecular weight region, which indicates that no homopolymer contamination was generated in the reactions, while PVC-0.50PEC and PVC-0.75PEC show two GPC peaks, indicating that homopolymer was produced in the reactions. In addition, the peak area of internally plasticized PVC materials decreased gradually with an increasing number of cardanol groups grafted onto the poly (vinyl chloride), which indicates that the highly branched internally plasticized PVC materials had been filtrated by the organic membrane in the dissolution process. The data for number-average molecular weight (*M*_n_), weight-average molecular weight (*M*_W_), and polydispersity indices are presented in Table 2. *M*_n_ and *M*_W_ of PVC materials increased gradually with greater numbers of cardanol groups grafted onto the PVC matrix. *M*_n_ of PVC, PVC-0.25PEC, PVC-0.50PEC and PVC-0.75PEC was 15,100, 21,200, 23,800 and 25,400 g/mol, respectively, which indicates that the internally plasticized PVC materials had been obtained.

### 3.3. Performances of Internally Plasticized PVC Materials

**Figure 5:**
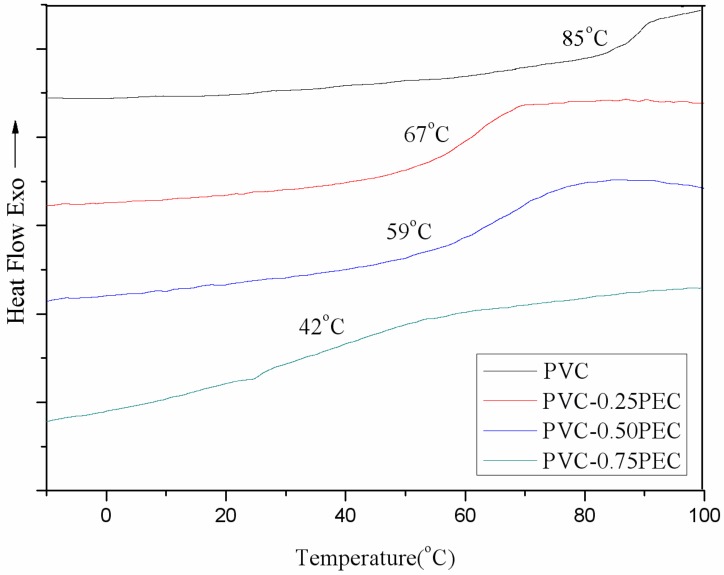
DSC curves of PVC and internally plasticized PVC materials.

The thermal properties of PVC, PVC-0.25PEC, PVC-0.50PEC and PVC-0.75PEC with different cardanol group contents were investigated using DSC and TGA measurements. As shown in Figure 5, the DSC curves of PVC and internally plasticized PVC materials with increased of cardanol group content did not present any melting peaks, indicating that all of the PVC materials had amorphous characteristics. A clear broadening of the *T*_g_ is observed, which indicates that the PVC and cardanol are not miscible. Additionally, none of the PVC materials exhibited any melting peaks, indicating their amorphous characteristics. *T*_g_ data for PVC, PVC-0.25PEC, PVC-0.50PEC and PVC-0.75PEC are summarized in Table 2, and were 85, 67, 59 and 42 °C, respectively. The decline in *T*_g_ with different contents of cardanol groups was mainly caused by the internal plasticizing effect of the cardanol groups. *T*_g_ is an important evaluation criteria in Mauritz and Storey’s mathematical models [31]. In this theory, plasticizing efficiency for reducing *T*_g_ can be controlled by structural features of the plasticizer, such as the branchiness of the side chains and the length of those chains. For the same molecular mass, branched plasticizers have higher plasticizing efficiency parameter values than linear structures. In this study, internally plasticized PVC macromolecules with higher branchiness side chains caused it to become a kind of internal plasticizer. The decrease in *T*_g_ illustrated that internally plasticized PVC materials induced a plasticizing effect on themselves. 

**Figure 6:**
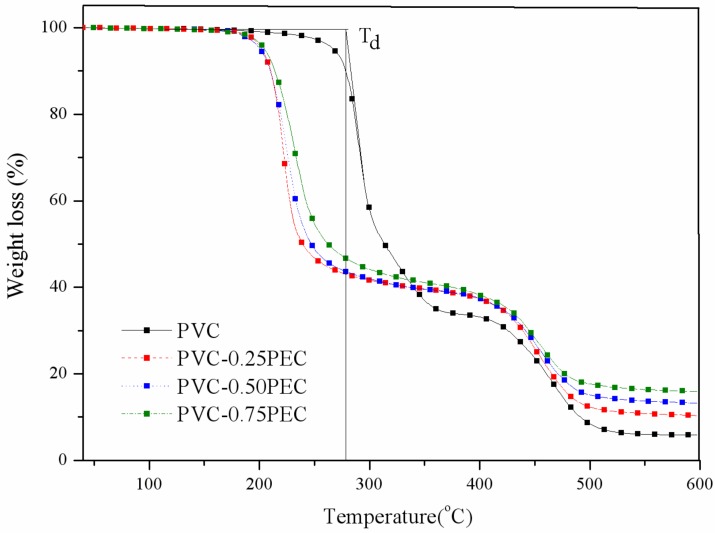
TGA curves of PVC and internally plasticzied PVC materials.

The onset degradation temperature (*T*_d_) and char residue are summarized in Table 2. As can be seen from Table 2, internally plasticized PVC materials had poorer thermal stability than PVC. The *T*_d_ of internally plasticized PVC materials decreased from 278.4 to around 210 °C with an increase in the number of cardanol groups grafted onto the PVC, which was caused by the unstable properties of azide groups under high temperature [18]. Char residue of PVC was 5.8% at 600 °C, the data reached 17.6% for PVC-0.75PEC. The reason for this is the fact that internally plasticized PVC materials have a higher relative carbon content than PVC, and that the thermal degradation process of internally plasticized PVC materials produces more char residue than PVC. Figure 6 presents the TGA curves of PVC and internally plasticized PVC materials with different contents of cardanol groups. PVC grafted with a variety of different functionalities showed a decrease in thermal stability below 220 °C, and increase in thermal stability above 220 °C. With an increased number of cardanol groups grafted onto the PVC chains, the thermal degradation of PVC-0.50PEC and PVC-0.75PEC at above 220 °C produced more char residue than PVC-0.25PEC. The char residue plays an important role in improving the thermal stability of internally plasticized PVC materials, because the char residue covers on the surface of internally plasticized PVC materials and prevents them from undergoing pyrolysis. One recent study has indicated that pyrolysis products of cardanol derivatives in gas phase include –OH– containing compounds, saturated hydrocarbons, CO_2_, CO, and aromatic derivatives; all of these compounds were burned and produced char residue [32]. Therefore, the potential degradation products of the new plasticizer do not have any adverse effects on humans or the surrounding environment. 

The common non-attached plasticizer based on cardanol acetate [6], epoxidized cardanol phenyl phosphate ester plasticizer [33], and epoxidized cardanol laurate [34] are easily leached from PVC in organic solvent, which is not conducing to make PVC products long-lasting and stable. The loss of plasticizers pollutes environments and produces a potential threat to human health. The internal plasticization strategy can effectively avoid the loss of plasticizer. The leaching tests of internally plasticized PVC films and PVC–DOP films were studied in n-hexane at 50 °C for 2 h. The results showed no migration in the leaching tests for internally plasticized PVC films, but 15.7% of the DOP leached from PVC–DOP films into n-hexane. These results indicate that the internal plasticization strategy was efficient for avoiding loss of plasticizers. 

### 3.4. Mechanism of Internal Plasticization

**Figure 7:**
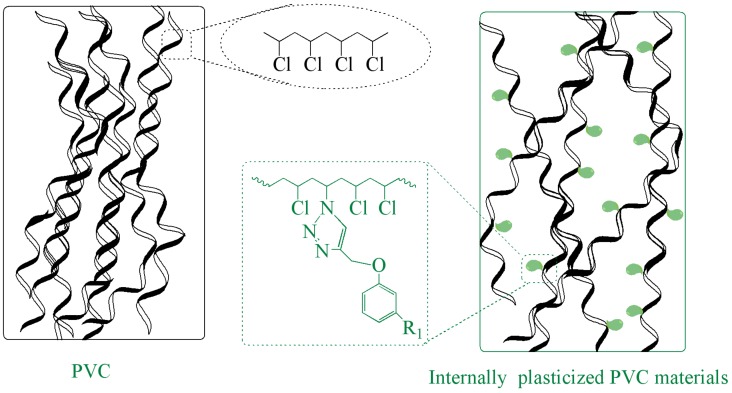
Structure of PVC and internally plasticized PVC materials under lubrication theory.

Traditional plasticization mechanisms include lubricity theory, gel theory, free volume theory, kinetic theories and mathematical models. Internal plasticization mechanisms can also be illustrated based on these theories. Kilpatrick [35] and others [36,37] developed lubricity theory. This theory holds that plasticizer, which acts as a molecular lubricant, allows the polymer chains to move freely over one another when a force is applied to the plasticized polymer [34]. Figure 7 shows the structure of PVC and internally plasticized PVC materials under lubrication theory. PVC chains cannot move freely, because these PVC chains without branched chains are tangled up. These molecular characteristics of polymer chains make PVC present as stiff and difficult to process. For internally plasticized PVC materials, cardanol groups grafted onto PVC chains can serve as a kind of molecular lubricant; this structure helps them move freely, causing internally plasticized PVC chains to present as flexible and easy to process. 

**Figure 8:**
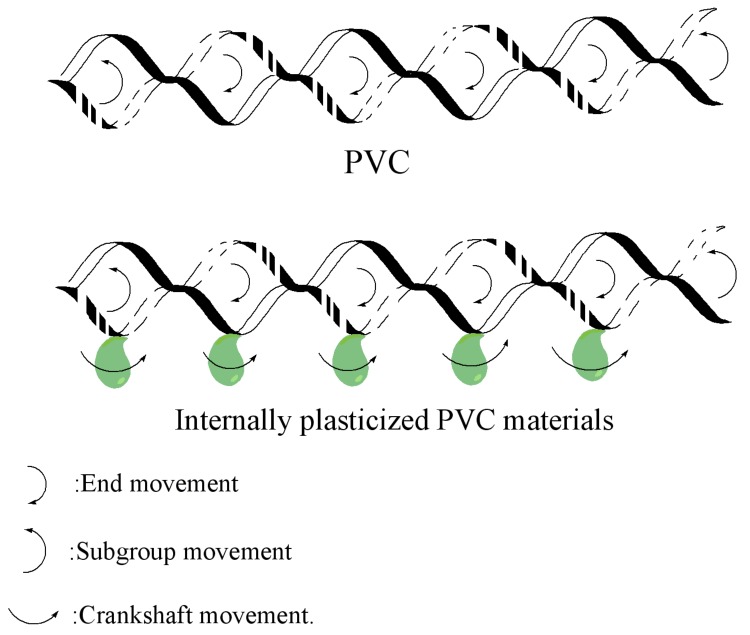
Three kinds of movement for chain-like macromolecules in free volume theory.

Free volume theory can be used to explain internal plasticization by evaluating the three kinds of movement for chain-like macromolecules: end movement, subgroup movement and crankshaft movement [38]. As can be seen from Figure 8, there are two kinds of movement for PVC macromolecules, because there are no branched chains on the chemical structure of PVC. Meanwhile, there are three kinds of movement for internally plasticized PVC macromolecules, because cardanol groups were grafted onto PVC. The grafting of cardanol groups onto PVC increases the distance and free volume of PVC chains and makes them easier to move. The increase in distance and free volume promotes the end movement, subgroup movement and crankshaft movement of internally plasticized PVC chains, causing the materials to present as flexible and easy to process. 

## 4. Conclusions

Internally plasticized PVC materials were synthesized by the grafting of PEC. The obtained PVC-0.25PEC, PVC-0.50PEC and PVC-0.75PEC exhibited efficient internal plasticization effects. Because cardanol groups grafted onto PVC chains is able to serve as a kind of molecular lubricant, the structure caused internally plasticized PVC chains to move freely, further increasing the distance and free volume of PVC chains and making them much easier to move than PVC. This will cause PVC materials to possess potentially flexible qualities, making them easy to process. The obtained internally plasticized PVC materials exhibited poorer thermal stability and more char residue than PVC. The *T*_g_ of the internally plasticized PVC materials reached 42 °C. No migration was found in leaching tests for internally plasticized PVC films, but 15.7% of DOP leached from PVC–DOP films into n-hexane. Therefore, this work provides a meaningful strategy for designing advanced PVC materials by introducing cardanol groups as branched chains.

## Figures and Tables

**Table 1:** Relative molecular mass and polydispersity indices of PVC materials.

PVC materials	Number average molecular weight (*M*_n_/g·mol^−1^)	Weight-average molecular weight (*M*_W_/g·mol^−1^)	Polydispersity indices
PVC	15,100	18,900	1.2
PVC-0.25PEC	21,200	26,700	1.2
PVC-0.50PEC	23,800	30,200	1.3
PVC-0.75PEC	25,400	31,900	1.3

**Table 2:** TGA and DSC data of PVC materials.

PVC materials	*T*_d_ (°C)	*T*_g_ (°C)	Char residue (%)
PVC	278.4	85	5.8
PVC-0.25PEC	211.3	67	10.0
PVC-0.50PEC	209.4	59	16.5
PVC-0.75PEC	208.6	42	17.6

## Supplementary Materials

The following are available online at http://www.mdpi.com/2073-4360/9/11/621/s1, Figure S1: FT-IR spectra of cardanol and PEC, Figure S2: ^1^H NMR of cardanol and PEC, Table S1: The composition of reactants.
