# Supplementary material for: Cardanol Groups Grafted on Poly(vinyl chloride)—Synthesis, Performance and Plasticization Mechanism
Source: Polymers (Basel). 2017 Nov 15;9(11):621. doi: 10.3390/polym9110621 (PMC6418606; doi:10.3390/polym9110621)
Supplement: Supplementary file 1 [file polymers-09-00621-s001.pdf]

Article

Cardanol Groups Grafted on Poly(Vinyl Chloride) - Synthesis, Performance and Plasticization Mechanism

Puyou Jia[1], Meng Zhang[1,2*], Lihong Hu[1,2], Rui Wang[3], Chao Sun[3], Yonghong Zhou[1*]

[1] Institute of Chemical Industry of Forest Products, Chinese Academy of Forestry (CAF); National Engineering Lab for Biomass Chemical Utilization; Key Lab on Forest Chemical Engineering, State Forestry Administration; and Key Lab of Biomass Energy and Materials, Jiangsu Province, 16 Suojin North Road, Nanjing 210042, P.R. China; yhzhou777@sina.com
[2] Institute of New Technology of Forestry, Chinese Academy of Forest (CAF), Beijing 100091, P. R. China;zhangmeng82@163.com
[3] Nanjing Forestry University, College of Materials Science and Engineering, 159 Longpan Road, Nanjing 210037, P.R. China; 641214952@qq.com
* Correspondence: yhzhou777@sina.com; zhangmeng82@163.com; Tel.: +86-025-85482520

Supporting informations

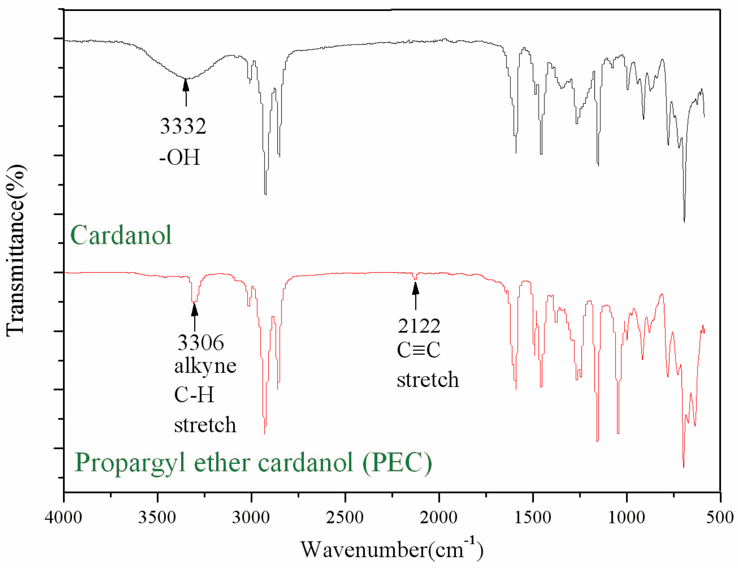

Figure.S1 FT-IR spectra of cardanol and PEC

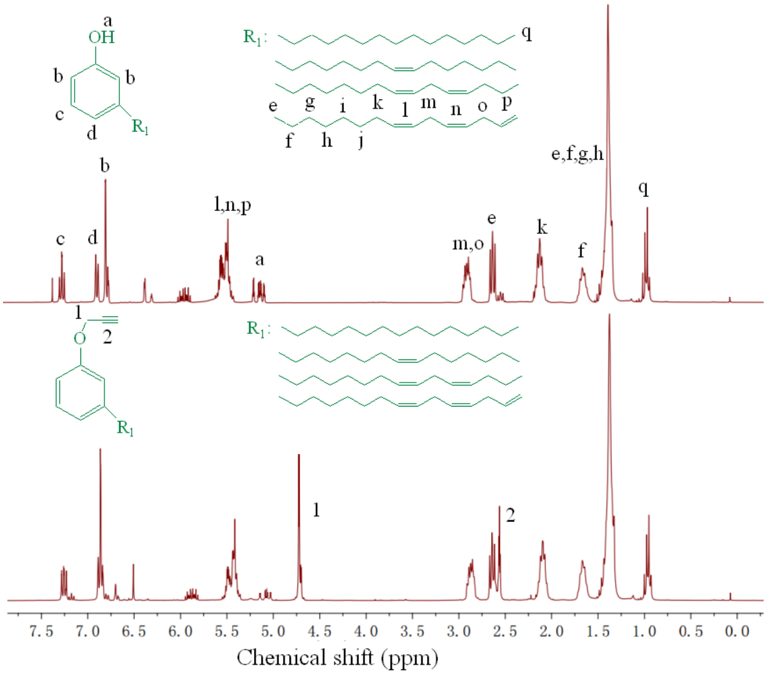

Figure.S2 ^{1}H NMR of cardanol and PEC

Table S1 shows the composition of reactants.

Internally plasticized PVC materials	PVC-N_3	PEC	Cuprous bromide	2, 2'-dipyridine
PVC-0.25PEC	1g(0.05m mol)	0.25g (0.73mmol)	0.21g (1.46mmol)	0.46g (2.92mmol)
PVC-0.50PEC	1g(0.05m mol)	0.50g (1.45mmol)	0.42(2.93mmol) cuprous bromide	0.92g(5.90mmol)
PVC-0.75PEC	1g(0.05m mol)	0.75g (2.19mmol)PEC	0.63g (4.38mmol) cuprous bromide	1.38g(8.85mmol
